# Comprehensive Analysis of the Influence of Soft Palate Inflammation in Brachycephalic Dogs with BOAS III

**DOI:** 10.3390/ani16020269

**Published:** 2026-01-15

**Authors:** Małgorzata Kandefer-Gola, Rafał Ciaputa, Izabela Janus-Ziółkowska, Kacper Żebrowski, Bartłomiej Liszka, Jakub Nicpoń, Stanisław Dzimira

**Affiliations:** 1 Department of Pathology, Division of Pathomorphology and Veterinary Forensics, Faculty of Veterinary Medicine, Wroclaw University of Environmental and Life Sciences, 50-375 Wroclaw, Poland; malgorzata.kandefer-gola@upwr.edu.pl (M.K.-G.); rafal.ciaputa@upwr.edu.pl (R.C.); izabela.janus-ziolkowska@upwr.edu.pl (I.J.-Z.); kacper.zebrowski@upwr.edu.pl (K.Ż.); 2 Department and Clinic of Surgery, Faculty of Veterinary Medicine, Wroclaw University of Environmental and Life Sciences, 50-366 Wroclaw, Poland; bartlomiej.liszka@upwr.edu.pl (B.L.); jakub.nicpon@upwr.edu.pl (J.N.)

**Keywords:** BOAS, brachycephalic syndrome, dogs, soft palate, inflammation, histopathology, degeneration

## Abstract

Brachycephalic obstructive airway syndrome is a group of abnormalities that primarily affect the upper respiratory tract, particularly in dogs—especially in English and French bulldogs, pugs, and Boston terriers. The description and the consequences of these anomalies are well known. We performed a detailed histopathological analysis of soft palate samples taken from brachycephalic dogs with BOAS III. We examined the impact of the severity and composition of inflammatory infiltrates on individual histological structures, such as mucosal membrane, serosal and mucosal glands, and muscles. The study was conducted on 50 samples of soft palate tissue collected from pugs and French bulldogs. The sections were then stained with haematoxylin and eosin (HE) and Masson–Goldner trichrome. In general, both lymphocytic and plasmocytic inflammation were observed. Plasmacytic inflammation was more commonly associated with more advanced changes, including glandular fibrosis, muscle degeneration, and waxy necrosis of the muscles. Therefore, inflammatory infiltration—particularly plasmocytic infiltration—is associated with more severe clinical symptoms and a poorer prognosis in BOAS III dogs.

## Simple Summary

Brachycephalic obstructive airway syndrome is one of the most common upper respiratory tract pathologies in dogs. The study evaluated the possible associations between the inflammatory reaction and progression of disease. An individual score for lesion grading was used. The results indicate that plasmacytic inflammatory infiltration is associated with greater histological changes in the soft palate tissues. Inflammation seems to play a significant role in the progression of clinical signs in brachycephalic obstructive airway syndrome.

## 1. Introduction

Brachycephalic obstructive airway syndrome (BOAS) is a group of abnormalities that primarily affect the upper respiratory tract. It is a lifelong condition, during which the chronic respiratory obstructive disease leads to a deterioration in quality of life. The diagnosis of BOAS is based on a few different characteristics. The most commonly reported anomalies include stenotic nares, aberrant turbinates, nasopharyngeal collapse, elongation and hyperplasia of the soft palate, everted laryngeal saccules, laryngeal collapse, and collapse of the left bronchus [1]. The breeds most predisposed to BOAS are English bulldogs, French bulldogs, pugs, and Boston terriers [2,3]. Based on the observed clinical signs, BOAS can be classified as follows: Grade 0 (BOAS-free and clinically unaffected; there are no clinical signs); Grade I (mild BOAS and clinically unaffected; the clinical signs are mild—nasal stertor during sniffing is observed and there will be no progression after exercise); Grade II (moderate BOAS and clinically affected; the clinical signs are mild to moderate—additionally, stridor and dyspnoea are evident at rest or with minimal activity); and Grade III (severe BOAS and clinically affected; moderate-to-severe clinical signs—dyspnoea is present; cyanosis or syncope may or may not be present, there will be an inability to exercise) [4,5,6]. Moreover, BOAS leads to hypoxia, which might also stimulate the generalised inflammatory response [7].

In brachycephalic dog breeds, elongation of the soft palate is a consequence of the shortening of the cranial bones—while these bony structures are reduced in length, the soft tissues remain unchanged [8,9]. The elongated soft palate can obstruct the laryngeal lumen, potentially leading to occlusion [10]. This elongated soft palate tissue is one of the most common causes of clinical manifestations of BOAS, such as coughing and snoring. A deterioration in health may lead to progression and more severe clinical symptoms such as hypoxia, stertor, dyspnoea, cyanosis, and syncope [6,11]. Affected dogs also exhibit exercise and heat intolerance. Further consequences may include tracheal and bronchial collapse, cor pulmonale, and pulmonary hypertension. The right ventricular dilation leads to heart failure. Additionally, the excessive aerophagia can result in gastric distension and flatulence, as well as increased gastrin secretion [12,13].

Clinical examination is supported by anamnesis; during anamnesis the breed and age of the dog are also quite important, as well as the clinical signs—also those which are observed by the owner at home [14]. During physical examination, stenotic nares are easy to diagnose. The prolonged soft palate and everted laryngeal saccules can be diagnosed only during general anaesthesia. Additionally, during the clinical examination, oedema and hyperaemia or congestion of the soft palate tissues and laryngeal area might be noticed. Moreover, thoracic radiography and cardiac electrocardiography are also recommended, especially in BOAS II and BOAS III [5,6,14].

During respiration, and specifically during inspiration, negative intrathoracic pressure is generated. In healthy dogs, this mechanism prevents airway collapse [15]. However, in brachycephalic dogs affected by BOAS, this negative pressure increases considerably during inspiration. This leads to chronic tissue vibration and stretching of the pharyngeal tissue, subsequent inflammation, and ultimately airway obstruction [10]. Because of that process, the soft palate tissues become longer and longer, which ultimately leads to even greater changes in the length of those structures. The chronic vibration of those tissues also leads to damage to tissue nerves in the soft palate, which can intensify the dyspnoea. The damage of the nerve endings also results in a reduction in muscle tension and—in effect—the response of the muscle tissue [7].

BOAS should be treated, especially when it becomes more severe with time or in the case of a life-threatening condition. Unfortunately, the most common curative method is surgical correction. Not every dog requires surgical correction, and the surgical procedure may be different in each patient. When an animal exhibits signs of exercise intolerance or heat intolerance, or there is difficulty in breathing, hypoxia, stertor, dyspnoea, cyanosis, or syncope, the shortening of the soft palate might be indicated. The surgery is usually based on the widening of the stenotic nares and shortening of the prolonged soft palate with the scalpel blade, scissors, or laser CO_2_ [16]. Palatoplasty is a surgical technique used for brachycephalic breeds, which plays a crucial role in improving clinical condition, even in middle-aged dogs [1,17]. The procedure involves shortening the elongated soft palate to restore airway patency and facilitate respiration. However, unfortunately, if secondary changes occur in the cardiovascular system—such as a remodelling of the right ventricle—they are irreversible. In that case, the secondary clinical signs associated with cardiovascular failure will not completely resolve.

The study aimed to perform a histopathological analysis of soft palates collected from dogs with BOAS III and to investigate the relationship between the predominant type and severity of inflammatory infiltrates, and the degree of epithelial hyperplasia, serous and mucous gland hyperplasia, muscular atrophy or hypertrophy, degenerative muscular changes, and fibrosis, across all structures.

## 2. Materials and Methods

### 2.1. Animals, Surgical Eligibility, and Operative Procedures

The study was conducted on 50 samples of soft palate tissue collected from brachycephalic dogs during palatoplasty procedures. In accordance with Polish law, standard surgical procedures performed on animal tissues do not require the approval of the Local Ethical Committee for Animal Experimentation [18]. The surgery formed a part of the therapeutic management of BOAS, with the aim of restoring airway patency. All operations were performed under general anaesthesia, with full consideration given to animal welfare. Informed consent for the surgical intervention and tissue collection for histopathological examination was obtained from the owners.

Brachycephalic dogs presenting with clinical signs of respiratory distress at the Veterinary Teaching Hospital underwent thorough clinical examination. Where elongation of the soft palate was confirmed, assessed, and surgical correction was deemed necessary, palatoplasty, and often nares correction, was performed.

Patients were eligible for surgery based on laryngoscopic examination. The study group consisted of 20 pugs and 30 French bulldogs, including 29 males and 21 females aged between one and four years. All dogs were classified as BOAS grade III. A total of 41 animals had also been diagnosed with stenotic nares. Moderate-to-severe stertor and stridor were observed, both of which could be heard without the use of a stethoscope. Reduced respiratory efficiency, manifested by increased inspiratory effort, was noted in all patients. All dogs exhibited exercise intolerance, as well as dyspnoea, an additional 15 dogs experienced syncope, and four dogs had a history of cyanosis.

The cases were selected based on inflammatory infiltrates; cases with predominant suppurative inflammation were excluded. The patients who had recently undergone antibiotic therapy were also excluded.

Prior to surgical intervention, haematological and biochemical blood tests were performed. The parameters routinely assessed during biochemical blood tests included ALT, AST, urea, creatinine, total protein, albumin, glucose, sodium, potassium, and chloride, as well as blood gas analysis. Cardiac electrocardiography (ECG) and thoracic radiography were also carried out. Respiratory acidosis was identified in twenty-eight dogs, whereas the remaining patients exhibited values within physiological reference ranges.

In the absence of any contraindications to anaesthesia, the patients were premedicated intravenously with methadone (0.2 mg/kg, Comfortan 10 mg/mL, Eurovet Animal Health B.V., Bladel, The Netherlands) and medetomidine (0.002–0.005 mg/kg, Sedator 1 mg/mL, Eurovet Animal Health B.V., Bladel, The Netherlands). General anaesthesia was then induced with propofol (titrated to effect at a dose of 1–5 mg/kg, Propofol Lipuro 10 mg/mL, B. Braun, Melsungen, Germany), following a period of pre-oxygenation lasting five minutes. The length of the soft palate and the severity of other BOAS symptoms were once again assessed at the time of endotracheal intubation.

Patients were connected to an inhalational anaesthesia machine (Mindray Wato-Ex 65 Pro), and general anaesthesia was maintained using sevoflurane (Sevoflurane, Baxter), which was vaporised in oxygen. If thoracic radiography excluded aspiration pneumonia, intravenous dexamethasone (0.5 mg/kg, Rapidexon 2 mg/mL, Eurovet Animal Health B.V., Bladel, The Netherlands) was administered to reduce postoperative oedema. Prior to surgery, amoxicillin and clavulanic acid (20 mg/kg, Synulox 140 + 35 mg/mL, Zoetis, Borgo San Michele, Italy) were administered subcutaneously. Any signs of intraoperative pain were managed with a fentanyl bolus (2 µg/kg, Fentadon 50 µg/mL, Eurovet Animal Health B.V., Bladel, The Netherlands), followed by a continuous infusion at a rate of 0.2 µg/kg/min.

During surgery, the elongated portion of the soft palate requiring correction was excised with a scalpel blade. The tissues were then sutured continuously using absorbable monofilament material, and the resected tissues were preserved in a formaldehyde solution for the histopathological analysis. Postoperatively, patients were maintained in an oxygen kennel with 40% oxygen and received a single intramuscular dose of buprenorphine (0.02 mg/kg, Bupaq Multidose, 0.3 mg/mL, Richter Pharma AG, Wels, Austria). All patients were discharged on the same day, provided that no post-extubation complications such as haemorrhage, respiratory distress, or inflammatory response were observed.

### 2.2. Histopathological Examination

For the histopathological examination, samples of the soft palate tissue (size 1–1.5 cm length and 0.4–0.9 cm width) were fixed in 10% formalin for 24 h, and transverse sections were obtained from these samples. The samples underwent standard histological processing involving dehydration through a graded series of alcohols, clearing in xylene, and embedding in paraffin wax. From the paraffin blocks, sections 3–4 µm thick were cut. These sections were then stained with haematoxylin and eosin (HE) and also with a Masson–Goldner trichrome.

The HE staining was used to evaluate the hyperplasia of the oral and nasopharyngeal epithelium, glandular hyperplasia (in both serosal and mucosal glands), muscular hypertrophy or atrophy, as well as muscular hyaline degeneration or waxy necrosis. The inflammatory infiltrates were also assessed based on the HE staining. The inflammatory infiltrates were evaluated. During the assessment of the inflammatory infiltration, its location and intensity were taken into account, as well as its cellular composition. The Masson–Goldner trichrome staining was used to evaluate the fibrosis around the glandular tissue and within the muscular layer. All of the above changes were assessed in five high-power fields (HPFs) at 400× magnification, corresponding to a total area of 2.37 mm^2^.

The histopathological examination was performed by a team of three experienced pathologists. During the analysis, epithelial hyperplasia and sub-epithelial inflammatory infiltrates were initially evaluated on both the oral and nasopharyngeal sides. Epithelial hyperplasia was graded as none (−), weak (+), moderate (++), or severe (+++). Inflammatory infiltrates of the epithelium were assessed based on their cellular composition and classified as either lymphocytic and/or plasmacytic. They were then graded using the same scale: none (−), weak (+), moderate (++), or severe (+++).

Subsequently, the presence of hyperplasia in the serous and mucous glands, as well as the presence of lymphocytic and/or plasmacytic inflammatory infiltrates between the glandular ducts, was evaluated. The fibrosis around the glandular structures was also assessed. All features were graded using the same four-point scale, as follows: none (−), weak (+), moderate (++), or severe (+++).

The final component of the histological assessment involved evaluating the muscle fibres. Changes such as atrophy, hypertrophy, hyaline degeneration (a pathological process where muscle fibres lose their normal structure, becoming swollen, glassy (hyaline), and losing cross-striations), and waxy necrosis (a form of coagulative necrosis characterised by a glassy appearance) were recorded. The presence of inflammatory infiltrates between muscle fibres and muscular fibrosis was also assessed. All of these features were graded as well as none (−), weak (+), moderate (++), or severe (+++).

### 2.3. Statistical Analysis

Statistical analysis was performed using Statistica 13.3 (TIBCO Software). Due to the non-parametric nature of the data, differences between groups were assessed using a Kruskal–Wallis ANOVA test. Associations between variables were analysed using Spearman’s rank correlation test. A significance level of *p* < 0.05 was adopted. Microphotographs were obtained using a Leica DM500 microscope (Leica Microsystems, Wetzlar, Germany) coupled with a Leica ICC50W camera (Leica Microsystems, Wetzlar, Germany).

## 3. Results

### 3.1. Epithelial Lesions

Mild epithelial hyperplasia was identified on the oral side of the majority of specimens, accompanied by the swelling of the basal cell layer and keratinocytes. Oedema was additionally detected immediately beneath the epithelium within the connective tissue. A mild, predominantly lymphocytic inflammatory response was also present. A moderate correlation was observed between the severity of epithelial changes and lymphocytic inflammation (*p* = 0.02; r = 0.42). No statistically significant associations were observed for the other evaluated parameters.

### 3.2. Glandular Lesions

Glandular hyperplasia involved both serous and mucous glands. The periductal and periglandular inflammatory infiltrates were composed predominantly of plasmacytes. Mild (+) glandular fibrosis was also observed. No significant correlation was demonstrated between glandular hyperplasia and the inflammatory response (*p* > 0.05). However, a significant association was observed between the degree of glandular fibrosis and the plasmacytic inflammatory component (*p* = 0.01; r = 0.36).

### 3.3. Muscular Lesions

Mild-to-moderate muscle atrophy was observed, whereas muscular hypertrophy was absent in the majority of samples. No statistically significant relationships were found between inflammatory infiltrates and muscle atrophy or hypertrophy. Degenerative changes, including hyaline degeneration and waxy necrosis, were frequently observed. A statistically significant correlation was identified between the severity of degenerative muscular changes and plasmacytic inflammation (*p* = 0.01; r = 0.35), as well as between the extent of necrotic alterations and plasmacytic inflammation (*p* = 0.01; r = 0.34). Despite the presence of moderate-to-severe muscle fibrosis, no significant associations were observed with the inflammatory infiltrates.

### 3.4. Other Lesions Not Taken into Account

**Figure 1:**
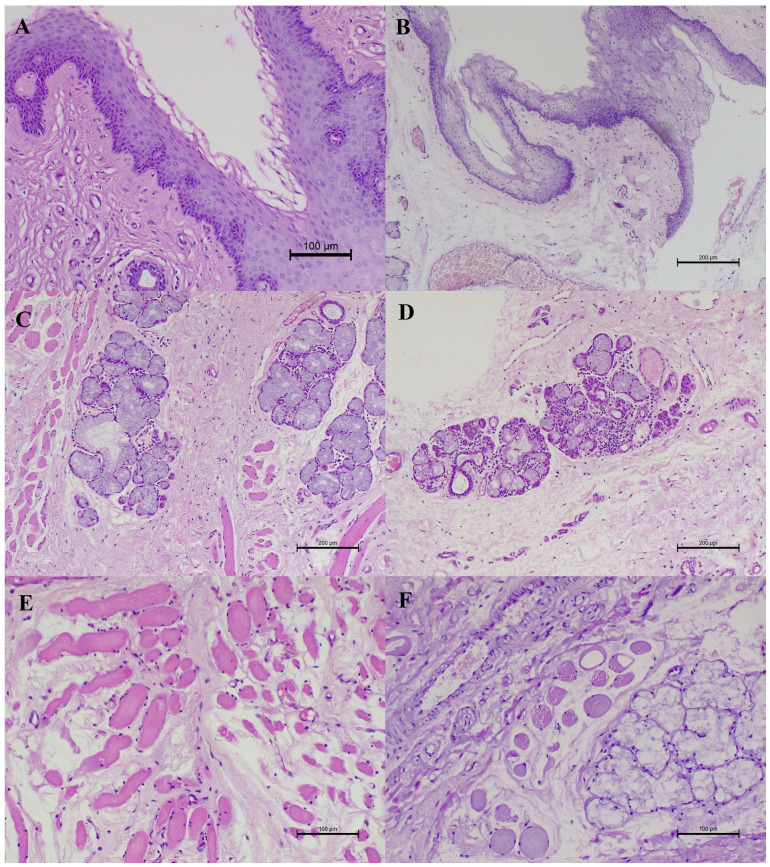
The histopathological changes in the soft palate. (**A**) The mild inflammation localised beneath the mucous membrane. HE, 200× magnification. (**B**) The average inflammation of the mucous membrane. The oedema of the epithelium and within the connective tissue. There is a mainly lymphocytic inflammatory response. HE, 100× magnification. (**C**) The average inflammation of the salivary glands. Plasmacytic infiltration predominates. HE, 100× magnification. (**D**) Severe inflammation of the salivary and individual serous glands. Predominantly plasma cell infiltration. HE, 100× magnification. (**E**) The average inflammation within the muscles. Visible hyaline degeneration. HE, 200× magnification. (**F**) The mild muscle inflammation. Areas of the waxy necrosis, hyaline degeneration, and oedema are visible. HE, 200× magnification.

**Figure 2:**
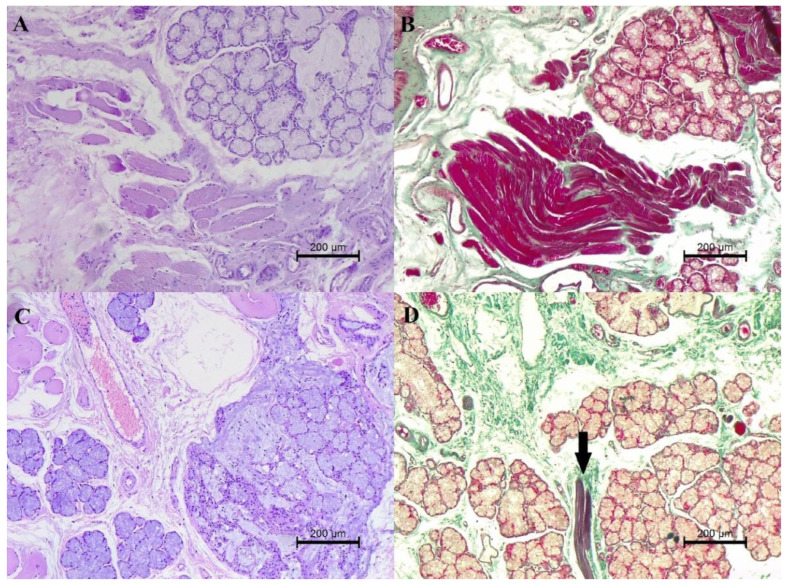
Histopathological changes in the soft palate—the comparison in the serial fragments. (**A**) The average fibrosis of salivary glands and muscles. HE, 200×. (**B**) The average fibrosis of salivary glands and muscles. Masson–Goldner trichrome, 200×. (**C**) Mild fibrosis of salivary glands and severe fibrosis of the muscles. HE, 200×. (**D**) Mild fibrosis of salivary glands and severe fibrosis of the muscles (arrows). Masson–Goldner trichrome, 200×.

Three samples also contained single mast cells or neutrophils, all in the superficial layers, but these data were not included in the statistical analysis. Table 1 and the accompanying figures present a summary of the obtained results [Figure 1 and Figure 2].

The results show the types of changes observed in individual histological structures, as well as the severity of these changes, indicating the number of cases (number of animals) and the percentage of samples in which a given change occurred.

## 4. Discussion

The anatomical and histological changes that occur in an elongated soft palate have been described several times previously. These changes are considered to be key factors in the thickening and elongation of the soft palate tissues [9]. Previous studies have investigated alterations to the muscles and nerves in BOAS grade I, focusing particularly on hypertrophy or atrophy, waxy necrosis, degenerative muscle changes, and the innervation of this tissue [9,19]. However, the significance of the severity and type of inflammatory response, as well as their influence on the soft palate tissues of dogs with BOAS grade III, have not yet been examined. In fact, the BOAS III constitutes the greatest challenge for clinicians and the most significant risk for patients. However, as with any study, this one also has certain limitations. No comparison with BOAS I or BOAS II was included, which was an intentional methodological choice. Previous research has primarily focused on identifying changes characteristic of BOAS I, where such alterations are the least pronounced. In BOAS III, the impact of inflammatory infiltration is considerably more apparent at the tissue level due to the greater severity and long-standing nature of the pathological changes.

During respiration, the negative intrathoracic pressure causes the stretching of the elongated soft palate tissue. In healthy animals, this phenomenon has no clinical relevance. However, in brachycephalic dogs, this effect has a significant impact on the muscle tissue, leading to its damage as a result of continuous mechanical stretching. Initially, the mechanical injury to the muscle causes haemorrhages within the tissue, followed by necrosis and inflammatory response. After some time, if the clinical symptoms do not resolve, the inflammation progresses with the activation of macrophages and fibroblasts, which ultimately leads to tissue repair and scar formation [20,21].

In the present study, an association was observed between hyaline muscle degeneration with waxy necrosis and the presence of plasmacytic inflammatory infiltrates. It is likely that this inflammation developed as a result of the necrosis occurring during the stretching of the soft palate tissue. Thus, the following cascade of events can be proposed: waxy necrosis primarily induces plasmacytic inflammation, which subsequently contributes to the hyaline degeneration of the muscles. Chronic muscle damage and the associated inflammation ultimately result in atrophy. Atrophy has also been reported by Arai et al. [19]; however, these studies did not evaluate the inflammatory response in soft palate tissues. The authors also suggested that negative intrathoracic pressure could be one of the contributing factors.

We demonstrated that lymphocytic inflammatory infiltrates predominated in the mucosal layer, while plasmacytic infiltrates were predominant in the glands and muscles. This pattern may be the result of different factors stimulating inflammation in these tissues. In the mucosa, the inflammatory infiltrate contributes to tissue thickening and oedema. In the glands, hyperplasia likely acts as a stimulus for inflammation. In the muscles, waxy necrosis induces plasmacytic inflammation. These findings are in line with human obstructive sleep apnoea syndrome (OSAS) research, which has reported similar observations in soft palate tissue studies [7,22,23].

The epithelial structure undergoes substantial changes, with microscopic studies primarily revealing hyperplasia and mucosal oedema [9]. The thickening of the mucosa is likely to be induced by snoring during respiration, with continuous tissue vibration leading to inflammation [24]. A similar process has been observed in humans with obstructive sleep apnoea syndrome [7]. Furthermore, the present study demonstrated a correlation between lymphocytic inflammation and the severity of mucosal changes. This suggests that, in dogs with BOAS grade III, snoring-induced soft palate vibrations stimulate the development of lymphocytic inflammation, exacerbating tissue oedema and thickening. A comparable relationship has also been reported in the mucosa of humans with OSAS, where the inflammatory infiltrate is predominantly composed of T lymphocytes [7,20].

Glandular hyperplasia was observed in the majority of examined tissues, affecting both mucous and serous glands. On the other hand, the inflammatory response does not appear to influence this developmental process. In humans, impaired respiration, snoring, and tissue vibrations induce glandular hyperplasia, and a similar mechanism is likely to occur in animals [25]. It is possible that glandular hyperplasia or changes in the glandular secretions trigger inflammation, which may subsequently lead to tissue fibrosis. Fibrosis and inflammation have also been reported in the hyperplastic salivary glands in older humans, in association with ageing or salivary gland disease [26,27].

In brachycephalic dogs, several different, independent factors affect the animal’s clinical condition and the possibility of deterioration in the clinical symptoms. The genetically transmitted feature of skull bone shortening was also compounded by the breeding methods used, including attempts to produce dogs with the shortest possible muzzles [6]. Unfortunately, selective breeding for extreme brachycephaly, a practice prioritising phenotypic traits over health, exacerbates the genetic predisposition to skull bone shortening, and potential owners often focus on the dog’s appearance rather than its health and welfare. The costs and morbidity of surgical treatment are a welfare concern for owners [28,29]. Airway oedema arising from ADAMTS3 dysfunction predisposes dogs to obstructed breathing, and these dogs were also found to have dilated lymphatic vessels. The relationship between morphological structure and genetic background is still not fully understood [28]. This is compounded by the effect of tissue stretching during breathing. In addition, longer soft palate tissues are subjected to greater vibrations. The stretching and vibration of these tissues during breathing also contributes to the haemorrhages and inflammatory response within them. All of the above processes also contribute to nerve damage, which causes a vicious cycle and leads to even greater damage. An additional factor is clinical symptoms, including hypoxia, which are known to stimulate the production of pro-inflammatory mediators [19,29]. On top of all this, there is also the negative effect of inflammation—i.e., hyperplasia or the atrophy of histological structures, as well as their degeneration, necrosis, and fibrosis. The continuous mechanical stimulation of tissues and worsening clinical symptoms cause increasingly severe changes in tissues, resulting in their fibrosis [30].

In our research, we have demonstrated the impact of inflammatory infiltration on changes in the histological image of soft palate tissues. With appropriately implemented therapy, it is possible to inhibit further stretching of the soft palate. Moreover, many brachycephalic dogs that appear clinically normal are, in fact, experiencing chronic hypoxia along with its systemic sequelae. Concurrent conditions associated with gastroesophageal reflux, sleep disturbances, and systemic hypertension further exacerbate the welfare implications for affected individuals [2]. Although inflammation was strongly associated with degenerative and fibrotic changes, the present study does not include molecular or mechanistic investigations. Therefore, the findings indicate correlation rather than causation. Future work incorporating immunohistochemistry, gene expression profiling, or cytokine analyses will be required to clarify the mechanistic role of inflammation in BOAS progression.

## 5. Conclusions

Inflammation appears to exert a significant role in the development and progression of BOAS III, especially plasmocytic inflammation in degeneration, necrosis and fibrosis of the muscle, as well as glandular fibrosis. The character of inflammatory infiltration varies between histological structures—lymphocytic infiltration predominates in the mucosa, while plasmacytic infiltration predominates in the muscle layer and within the glandular part. It is worth mentioning that inflammation differed in the various histological layers, as it was caused by different processes that were independent of each other but overlapped. The stimulation of inflammation and the subsequent maintenance or exacerbation of this process by intensifying clinical symptoms cause a vicious cycle of effects. Clinical signs, including stertorous breathing, dyspnoea, excessive negative intrathoracic pressure, and snoring, lead to the continuous vibration and stretching of the tissues, which in turn exacerbate the pathological process.

Understanding the role of inflammation may support the development of more effective therapeutic strategies and improved prognosis assessment in dogs with BOAS. BOAS III therapy, even surgical, does not always result in a significant improvement in clinical condition. The implementation of anti-inflammatory therapy may stop the progression of the stretching of the soft palate. The surgical and non-surgical management of BOAS patients is essential.

We acknowledge that the present study has certain limitations, which is why we plan to broaden its scope in future work. Advancing our understanding of these pathophysiological mechanisms will necessitate future investigations that specifically assess tissue compliance and delineate the respective roles of B- and T-lymphocyte populations within the affected tissues. Planned research activities include the extension of the current methodology to incorporate immunohistochemical analyses capable of differentiating between B- and T-cell subsets. Furthermore, we intend to evaluate the robustness of the observed correlations across BOAS I and II, which will help determine whether our conclusions will be identical to those in BOAS III.

## Figures and Tables

**Table 1:** The histopathological findings in 50 dogs with BOAS III—the summary of the evaluated samples.

	None (-)	Weak (+)	Moderate (++)	Severe (+++)
Epithelial hyperplasia	3 (6%)	27 (54%)	13 (26%)	7 (14%)
Sub-epithelial lymphocytic inflammation	9 (18%)	35 (70%)	5 (10%)	1 (2%)
Sub-epithelial plasmocytic inflammation	35 (70%)	7 (14%)	7 (14%)	1 (2%)
Glandular hyperplasia	2 (4%)	12 (24%)	15 (30%)	21 (42%)
Glandular lymphocytic inflammation	17 (34%)	25 (50%)	5 (10%)	3 (6%)
Glandular plasmocytic inflammation	6 (12%)	12 (24%)	19 (38%)	13 (26%)
Glandular fibrosis	3 (6%)	29 (58%)	6 (10%)	12 (24%)
Muscular atrophy	7 (14%)	17 (34%)	16 (32%)	10 (20%)
Muscular hyperplasia	30 (60%)	7 (14%)	11 (22%)	2 (4%)
Muscular hyaline degeneration	0 (0%)	11 (22%)	14 (28%)	25 (50%)
Muscular waxy necrosis	8 (16%)	23 (46%)	12 (24%)	7 (14%)
Muscular lymphocytic inflammation	20 (40%)	28 (56%)	2 (4%)	0 (0%)
Muscular plasmocytic inflammation	28 (56%)	17 (34%)	3 (6%)	2 (4%)
Muscular fibrosis	2 (4%)	10 (20%)	17 (34%)	20 (40%)

## Data Availability

The datasets used and/or analysed during the current study are available from the corresponding author upon reasonable request.

## Abbreviations

The following abbreviations are used in this manuscript:

BOAS	brachycephalic obstructive airway syndrome
ALT	alanine transaminase
AST	aspartate transferase
ECG	cardiac electrocardiography
HE	haematoxylin and eosin
HPF	high-power field
OSAS	obstructive sleep apnoea syndrome
ADAMTS	a disintegrin and metalloproteinase with thrombospondin motifs
